# Countrywide survey on utilization of medical devices by GPs in Hungary: advantages of the cluster-practice model

**DOI:** 10.1017/S1463423621000372

**Published:** 2021-06-29

**Authors:** Katalin Dózsa, Fruzsina Mezei, Tamás Tóth, Ábel Perjés, Péter Pollner

**Affiliations:** 1 Health Services Management Training Centre, Semmelweis University, Budapest, Hungary; 2 National Public Health Center, Budapest, Hungary; 3 Omron Hungary, Budapest, Hungary; 4 Institute of Digital Health Sciences, Semmelweis University, Budapest, Hungary; 5 MTA-ELTE Statistical and Biological Physics Research Group, Eötvös Loránd Research Network (ELKH), Department of Biological Physics, Eötvös University, Budapest, Hungary

**Keywords:** cluster-practice, infrastructure, medical devices, NCD, primary care development

## Abstract

**Background:**

Expectations towards general practitioners (GPs) are continuously increasing to provide a more systematic preventive- and definitive-based care, a wider range of multidisciplinary team-based services and to integrate state-of-the-art digital solutions into daily practice. Aided by development programmes, Hungarian primary care is facing the challenge to fulfil its role as the provider of comprehensive, high quality, patient-centred, preventive care, answering the challenges caused by non-communicable diseases (NCDs).

**Aim:**

The article aims to provide an insight into the utilization of simple, digital, medical devices. We show the relationship between the primary health care (PHC) practice models and the used types of devices. We point at further development directions of GP practices regarding the utilization of evidence-based medical technologies and how such devices support the screening and chronic care of patients with NCDs in everyday practice.

**Methods:**

Data were collected using an online self-assessment questionnaire from 1800 Hungarian GPs registered in Hungary. Descriptive statistics, Wilcoxon’s test and *χ* ^2^ test were applied to analyze the ownership and utilization of 32 types of medical devices, characteristics of the GP practices and to highlight the differences between traditional and cluster-based operating model.

**Findings:**

Based on the responses from 27.7% of all Hungarian GPs, the medical device infrastructure was found to be limited especially in single GP-practices. Those involved in development projects of GP’s clusters in the last decade reported a wider range and significantly more intensive utilization of evidence-based technologies (average number of devices: 5.42 versus 7.56, *P*<.001), but even these GPs are not using some of their devices (e.g., various point of care testing devices) due to the lack of financing. In addition, GPs involved in GPs-cluster development model programmes showed significantly greater willingness for sharing relatively expensive, extra workforce-demanding technologies (*χ* ^2^ = 24.5, *P*<.001).

## Introduction

Health systems around the world face several challenges due to changing demographic, cultural, economic and technological conditions, and Hungary is no exception. Hungary’s position in terms of lifestyle-related risk factors consequent non-communicable diseases and avoidable mortality lags behind the global average. The Hungarian health care system is relatively underfunded in the share of Gross Domestic Product (GDP) – compared to the European Union (EU) average – and despite relevant investments of the last decade into primary health care (PHC) financing, the health care is still hospital centred. Emigration of health care workers resulting in labour shortages and the ageing of medical professionals are further challenges in the region. As the first contact with the health care system, primary health care teams are in a unique position to improve health outcomes and reduce unnecessary hospitalizations, associated costs and adverse health outcomes by facilitating people-centred care (OECD and EU, 2020). Strengthening primary care has been identified by OECD (2020) as an effective policy tool thrive for an efficient, people-centred and equitable health system. Health at a Glance Report series of Organisation for Economic Co-operation and Development (OECD) addressed the weakest points of the Hungarian single-practice-based PHC service model and recommended to invest in preventive and more definitive care provided by multidisciplinary teams [general practitioners (GPs)-clusters instead of single practices] (OECD and EU, 2016).

Recent international data show that too many patients with chronic conditions do not receive the recommended preventive care. Despite contrary endeavours in policies and development programmes, Schafer *et al.* (2016) report that involvement in curative care has increased all over Europe over the past decade, while involvement in preventive activities has decreased by 13% on average between 1993 and 2012. Hungary saw one of the most significant decreases of more than 50% in preventive activities. In addition to currently prominent curative care, more emphasis needs to be placed on disease prevention and health promotion, with focus on future health services addressing the major burden of chronic disease, risk factors and reducing inequalities in both European and Hungarian context. Based on statistics of EuroStat and OECD, it is clear that Hungary has to disseminate the evidence of recently completed GPs-cluster pilot programmes’ and continue investing into the systematic, evidence-based screening, risk-management and chronic-care services of primary health care. That requires a proper amount of trained health-care professionals, modern primary health care centres for multidisciplinary teams, equipment and novel systematic performance-based financing instead of the present capitation-based financing.

In today’s climate, an efficient primary care system needs to leverage all functionalities offered by available medical devices and digital technologies to support health outcomes and health-related activities, facilitating the uptake of cost-effective preventive activities instead of a costly curative model of care. Striving for better health outcomes and economic necessity means a shift towards new models of people-centred primary health care based on teams and networks. A Swiss–Hungarian and several EU co-funded development programmes have been initiated in Hungary in the last decade to facilitate the high quality and coordinated preventive function of primary care. The programmes aided the formation of multidisciplinary GP clusters in vulnerable regions of the country, the completion of targeted population screening programmes, health education and medical device and digital technology procurements (Ádány *et al.*, 2013). Two state-funded development programmes followed these initiatives, and for today more than 1000 out of the 6503 GP services in Hungary were involved in GPs-cluster or consortium model programmes. The introduction of a state-funded systematic multidisciplinary GPs-cluster model is on the way, expected from 2022. The relevant Government Decree was issued in February 2021. Inevitably, the role of primary care in Hungary is becoming increasingly significant and needs to stand as a solid cornerstone for an efficient health care system.

Due to previous efforts, Hungarian primary care is in transition, facing the challenge to fulfil its role as the provider of comprehensive, high quality, patient-centred, preventive care. The growth of GPs’ responsibilities, the introduction of novel preventive and chronic-care services puts the currently existing organizational and infrastructural framework under pressure. Health care management and health service research have frequently focussed on the role of infrastructure and adequate equipment to assess the quantity and quality of health care services (Guilbert, 2006). It is obvious that adequate physical infrastructure, medical devices and IT infrastructure are crucial for preventive and curative medical services. The role of health care facility infrastructure as a major component of a health care system must not be underestimated. Infrastructure constitutes one of the components of the World Health Organization (WHO) Alliance for Health Policy and Systems Research’s six building blocks of health care systems (Adam and De Savigny, 2012). The collection and analysis of data on facility infrastructure enable policymakers to detect and eliminate infrastructural deficiencies to improve the performance of a health care system. This leads to better services, for example, by assuring the availability and functioning of the required technical medical equipment for preventive and screening purposes and to the improvement of health outcomes in the population as a consequence of improving accessibility, availability and quality of health services due to good facility infrastructure (Scholz *et al.*, 2015).

Point of care diagnostic and therapeutic devices are important elements of a 21st century primary care provider. Using these medical devices can contribute to the early detection of various diseases, and can improve the cost-effectiveness of the care, for example using C-reactive protein (CRP) tests for respiratory tract infection to reduce antibiotic use (Hunter, 2015) or using point of care screening for identifying patients at risk for chronic obstructive pulmonary disease (COPD; Thorn *et al.*, 2012). The relevance of primary care CRP testing was recognized by several policymakers and included in evidence-based guidelines amongst others in the UK (NICE, 2019) and the Netherlands (Verheij *et al.*, 2011). The recent development of medical devices enabled to move several routine diagnostic tests from the central laboratories to the GPs’ offices, however, the financing should follow these changes and financial incentives should be developed in order to cover the additional costs of point of care diagnostic. The Danish regulators have developed a new fee-for-service reimbursement method for haemoglobin A1c (HbA1c) testing for diabetes patients in general practice (Kristensen *et al.*, 2017). In addition to the investment and operational costs, barriers include infrastructural shortages (layout of the facility, placement of the devices), limited time during the consultations, increased workload and resistance to change (Johnson *et al.*, 2018).

### Objectives

The article aims to provide an insight into routinely used medical devices, and to point at further development and educational directions of GP practices regarding the utilisation of guideline-indicated medical technology in everyday practice. The presented data are to serve the improvement of preventive and definitive health care services at PHC level through technical infrastructure, extending competencies of PHC professionals and support decision-makers in planning targeted budget and incentives for medical device investments and utilization. Results of past development efforts and lessons for follow-up development steps supporting the structural transition of a hospital centred health care system to an integrated, efficient, effective, accessible, digitalised, multidisciplinary team-based primary health care centred system are identified and discussed.

## Methods

The article is based on data from the Hungarian PHC Infrastructure Study that was completed in the framework of the EFOP 1.8.0 – VEKOP 17-2017-00001 “Professional methodological development of the health care system” programme. The study involved 2358 GPs out of 6503 (36%), 1614 health visitors out of 5019 (32%), and 1060 dentists out of 2824 (37%) registered in Hungary in 2020. Data were collected about the size and condition of the buildings, the available and required rooms, the cooperation of other specialities and the utilization of info-communication technologies and medical devices as part of a complex survey of the primary care infrastructure. Separate questionnaires were developed for GPs, primary care dentists and health visitors. The focus of this article is the assessment of ownership and utilization of medical devices taking into account data collected from the GP practices only.

To aid the development of an adequate, focussed questionnaire, focus group interviews were performed with GP specialist experts. Once enough input was gathered and the list of questions was finalized, the questionnaires were validated by practising GPs. The questionnaires were disseminated using the LimeSurvey platform, which enabled using logical rules of questioning, that is, different options were presented based on the previous answers. The link to the questionnaire was sent in an email to all registered 6503 Hungarian GPs at the end of February 2020. Due to the start of the COVID-19 epidemic in Hungary, the initial response rate was relatively low, therefore, follow-up requests were sent in the middle of April. With this approach, the response rates were doubled by the end of June.

Thirty-two medical devices were investigated in the questionnaire (listed in Table 1). The investigated tools were chosen based on evidence-based recommendations of international medical societies and European bodies. Some devices are indispensable for providing state-of-the-art, effective and efficient preventive services, early diagnosis of chronic conditions and adequate care to decrease premature death and already in the guidelines and national minimum standards regarding the medical device and medical technology ownership (Farrance, 2014; Hopstaken *et al.*, 2015; NICE, 2019).

**Table 1.** Comparison of the medical devices owned by single practices and GPs clusters

Device name	Ownership ratio (%)			*χ* ^2^	*P*-value
	Overall	Single practice	GP cluster		
Pulse oximeter	82.3	80.4	89.9	16.144	<.001*
ABPM	48.9	43.0	72.1	91.629	<.001*
Take-home digital blood glucose meter	42.7	37.4	64.1	78.546	<.001*
Calibrated tuning fork	39.2	38.0	43.3	3.0559	.08
Take-home digital blood pressure meter	38.9	33.1	62.3	97.178	<.001*
Automated external defibrillator	38.0	37.1	42.1	2.75	.10
TENS device	38.3	36.4	35.0	0.1642	.69
Pharmaceutical atomizer/inhaler	36.1	34.4	33.8	0.0143	.90
Autoclave	27.3	28.2	23.1	3.297	.07
Bioptron lamp	23.6	23.4	22.8	0.016	.90
Spirometer	22.9	19.5	37.1	46.844	<.001*
Germicidal lamp	20.0	20.6	18.4	0.672	.41
Ankle-arm index gauge	16.3	11.8	34.7	103.64	<.001*
Holter ECG	15.8	11.7	31.5	80.02	<.001*
Urine testing device	15.5	14.6	18.7	3.147	.08
CRP PoCT	13.5	12.5	17.5	5.3454	.02
Trans-telephone ECG	13.2	9.3	30.0	99.334	<.001*
INR PoCT	11.7	11.4	13.1	0.549	.46
Physiotherapy ultrasound device	6.6	6.4	7.4	0.293	.59
WIWE mobile ECG device	5.7	4.8	9.2	9.072	.003*
Exhaled carbon monoxide(CO) meter	5.4	2.9	15.7	85.71	<.001*
Diagnostic ultrasound device	5.1	5.1	5.3	0.002	.96
Haemoglobin PoCT	4.4	3.9	6.5	3.857	.049*
D-dimer PoCT	3.8	3.5	5.3	2.0717	.15
HbA1C PoCT	3.3	3.1	4.5	1.224	.27
apneABPM	2.9	2.6	4.2	1.899	.17
PSA PoCT	1.7	1.5	3.0	2.77	.10
Lymphatic massage device	1.6	1.7	1.2	0.16	.69
iFOBT device	1.4	1.3	2.1	–	–
Shock wave therapy device	1.3	1.4	1.2	–	–
Blood gas analyzer	1.0	1.1	0.6	–	–
NT-pro-BNP device	0.7	0.8	0.6	–	–

GP = general practitioners; ABPM = ambulatory blood pressure monitoring; TENS = transcutaneous electrical nerve stimulation; CRP = C-reactive protein; PoCT = Point of care testing; INR = international normalized ratio; HbA1C = haemoglobin A1c; PSA = prostate-specific antigen; iFOBT = immunologic fecal occult blood test; NT-pro-BNP = N-terminal pro btype natriuretic peptide. The two types of practices were compared by the *χ* ^2^ test. Significant differences are marked with *.The test was not applicable to the last four devices due to the low-case numbers.

The questions regarding ownership, usage and opinion about medical devices included the following:

- In addition to the national minimum standard devices^1^, what additional medical devices do you own and how frequently do you use them? (choice of 32 devices – see Table 1)
- What are the reasons for the rare usage of a medical device? (if usage was indicated as rare or not used in the previous question for a medical device)
- What do you think about the medical devices listed? (essential, useful, not necessary in the GP practice)
- Would it require additional funding to operate the additional devices?
- Can you imagine sharing some high-value devices with other GP practices?

The National Health Insurance Fund identifier (“NEAK code”) and the postcode of the practice were collected for identification purposes. Using the “NEAK code” of respondents, answers were combined with the National Health Insurance Fund database, allowing analysis of additional characteristics, for example, practice type or geographical location without having to retain the information via the questionnaire. This allowed for a faster fill-out time and ensured data validity. The postcode was used for validating the answers. The LimeSurvey platform did not allow for the preliminary connection of the practice database, so it was not possible to predefine a list of practice codes to select from. The manual entry of the NEAK code caused some data quality issues, especially because both the financing agreement and the operating licence use nine-digit codes in Hungary which resulted in occasional confusion for the respondents.

The first step of the analysis was a data cleaning process on the results exported from LimeSurvey. Based on the unique IDs, the duplicate answers were deleted. The more complete answer (or the one with the later timestamp if the same number of questions were answered) was used for the analysis. The NEAK code and the postcode were double-checked with the NEAK database, and the differences were examined one by one.

The statistical analysis of the data was performed using Microsoft Excel and the RStudio. Descriptive statistics, Wilcoxon’s test and *χ* ^2^ test were applied. A major limitation of the data gathering methodology is the usage of self-reported questionnaires as respondents tend to be biased and report more optimistic results (Stone *et al.*, 1999).

## Results

### General characteristics of the responses

The raw response number was 2226 complete and 3822 partial filling. After removing the duplicates and the answers without significant information (e.g., those which were stopped after completing the identifier data), 2358 responses remained from which 1670 were complete. Precisely 1800 respondents (27.7% of all Hungarian GP practices) answered the questions about the medical devices, so these were used for the analysis presented in this article.

Precisely 1600 respondents entered a valid NEAK identifier so we could link these data to the NEAK database. These included 802 adult, 386 paediatric and 412 mixed practices. About 337 of the respondent GPs are participating in multidisciplinary GP clusters.

### Ownership of the devices

The average and the standard deviation of the number of medical devices owned by the GPs are 5.8 ± 3.66 while a median of 5. Forty-seven practices do not have any of the 32 devices listed, with three practices stating that they own all of them. Only 10% of the practices have more than 10 of the devices listed, in addition to the standard minimum devices. The single GP practices and the members of GPs’ clusters were compared using Wilcoxon’s test and the results show that the latter have a significantly larger number of medical devices (the average is 5.42 versus 7.56, *P*<.001). The results are also significant if the different types of practices are compared: the average number of devices per practice is 4.98, 5.84 and 6.86 for paediatric, adult and mixed practices, respectively. The results of Wilcoxon’s test are shown in Table 2.

**Table 2.** Pairwise comparison of the number of medical devices owned by different types of GP practices (adult, paediatric and mixed)

Practice type	Avg1	Avg2	*P*-value
Adult versus paediatric	5, 85	4, 98	<.001
Adult versus mixed	5, 85	6, 86	<.001
Paediatric versus mixed	4, 98	6, 86	<.001

The table depicts the average numbers of the devices and the result of Wilcoxon’s test. All *P*-values are significant.

We have calculated the ownership ratios for each device and practice type. In most cases, the lowest ratio was found by the paediatric practices, and the mixed practices own slightly more devices than the adult ones. However, some of the devices showed significantly different patterns:

- pharmaceutical atomizer/inhaler: two-third of the paediatric practices and a quite low number of adult practices own it (P: 67.9%, A: 18.6%, M: 37.3%);
- CRP point of care testing (PoCT): three times as much paediatric practices own it as the mixed practices, and a surprisingly low number of the adult practices (P: 35%, A: 3.7%, M: 11.1%);
- Haemoglobin (Hgb) PoCT: more common in paediatric practices, rarely present in adult ones (P: 9.8%, A: 1.7%, M: 4.4%);
- urine testing device: more common in paediatric practices (P: 24.1%, A: 12.2%, M: 14.3%);
- germicidal lamp: more common in paediatric practices (P: 28.8%, A: 16.7%, M: 19.6%);
- automated external defibrillator: more common in mixed practices, and only a few paediatric practices own it (P: 13.5%, A: 40.1%, M: 57.9%); and
- autoclave: more common in mixed practices, the adult and paediatric practices own it it similar ratio (P: 24.4%, A: 24.0%, M: 41.4%).

The most popular device to own is the pulse oximeter which is reported to be owned by 82% of the responding GPs. Seven more devices are owned by between half and one-third of the respondents: ambulatory blood pressure monitoring (ABPM) (49%), digital blood glucose meter for patients (43%), calibrated tuning fork (39%), digital blood pressure meter for patients (39%), transcutaneous electrical nerve stimulation (TENS) device (36%) and pharmaceutical atomizer/inhaler (34%). Less frequently owned devices include PoCT devices [Hgb, D-dimer, HbA1c, prostate-specific antigen (PSA), N-terminal pro b-type natriuretic peptide (NT-pro-BNP), immunologic fecal occult blood test (iFOBT), blood gas analyzer], apneABPM, lymphatic massage and shock wave therapy device with less than 5% of ownership amongst GPs each. Table 1 shows the detailed results on ownership sorted by frequency and including the differences between single practices and GPs clusters. The statistical evaluation was made using the *χ* ^2^ test. There are 11 types of devices that are significantly more common amongst the members of GPs’ clusters; the remaining devices did not show a significant difference.

### Utilization of the devices

Owning a device does not automatically mean that it is used in daily practice, so respondents were asked to rate the usage frequency of the devices they own. The answers were grouped into two categories: frequent usage (multiple times a day or multiple times a week) and rare usage (a few times a month or not at all).

**Figure 1.**
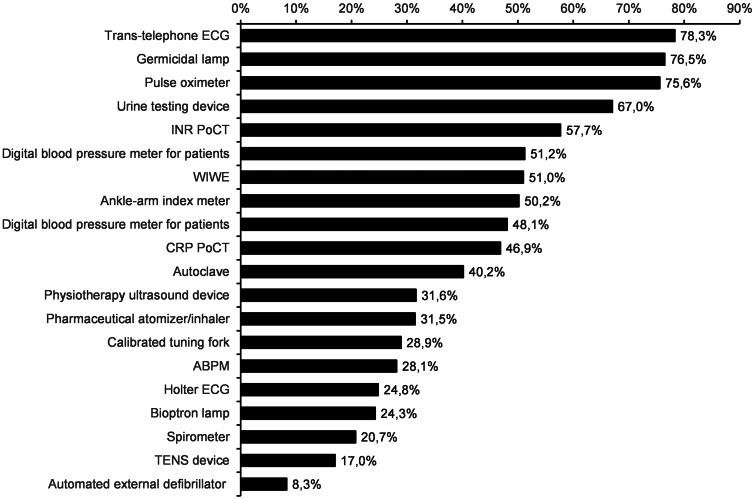
The ratio of general practitioners (GPs) reporting frequent usage of the investigated medical devices. Data are shown in the proportion of ownership. Only those devices are presented which are owned by at least 100 responding GPs

The average and the standard deviation of the number of frequently used devices are 2.4 ± 2.6, the median is 2. The most frequently used device is the pulse oximeter (1128 practices use it often). It is much higher than the next devices: take-home digital blood glucose meter (371), take-home digital blood pressure meter (360), germicidal lamp (276) and ABPM (247). In the proportion of the ownership, the most commonly used devices are the following: trans-telephonic ECG (79% of owners use it frequently), germicidal lamp (77%), pulse oximeter (76%), urine testing device (67%) and international normalized ratio (INR) PoCT (57%). Out of 1800 practices, there are 203 that use all their devices frequently. The detailed results are shown in Figure 1 (only those devices are presented which are owned by at least 100 responding GPs).

The difference between single and cluster practices is significant for the following three devices: Ankle-arm index meter (frequently used by 41.2% of single practices and 63.2% by cluster members, the *P*-value of the *χ* ^2^ test is <.001), CRP PoCT (43.9% versus 55.9%, *P*<.001) and ABPM (25.3% versus 35.4%, *P* = .004).

Most practices (1550) use one or more devices only rarely or not at all. The average and the standard deviation of the number of rarely/not used devices are 3.4 ± 2.8, the median is 3. The less frequently used devices in absolute numbers include the following: ABPM (638), automated external defibrillator (635), TENS device (541), calibrated tuning fork (504) and pharmaceutical atomizer/inhaler (424). In the proportion of the ownership, the following devices are used less frequently: Automated external defibrillator (92% of the owners), iFOBT PoCT (84%), HbA1c PoCT (83%), TENS device (83%), D-dimer PoCT (83%). These three PoCT devices are owned only by a small number of practices (25–69).

In summary, 41.8% of all devices are frequently used (on average 2.4 devices per practice) whereas 58.2% are rarely used (on average 3.4 devices per practice).

The most common reason for not or rarely using a device – according to the respondent – is that there are lack of patients requiring its usage on a daily basis. There are some devices that require too much time to use according to the respondents (e.g., ABPM, Holter-ECG, TENS device). The main reason for not using the various PoCT diagnostic devices is their high operational costs.

### Sharing medical devices

For a number of expensive devices, it would be reasonable to purchase and share usage amongst multiple practices. The willingness of the respondents to do so was measured, and results show that GPs working in a GP cluster are more likely to share devices with other colleagues (Table 3). Using *χ* ^2^ test the difference is significant (*χ* ^2^ = 24.5, *P*<.001). The collaboration would even be possible for single practices as many Hungarian GPs (especially in medium-sized and bigger settlements) are working in shared buildings provided by the local municipality.

**Table 3.** Comparison of the willingness to share medical devices amongst single-practice GPs and those participating in a GPs cluster

		Not participating in a GP cluster	Participating in a GP cluster
Ready to use devices together	Yes	842 (59%)	248 (73.8%)
	No	584 (41%)	88 (26.2%)

### Opinions about the devices

The respondents were also asked about their perception of the usefulness of the devices. The pulse oximeter was rated as the most useful: almost 70% answered “Required, should be part of the national minimum standard”. The second most useful device was the automated external defibrillator with 49% voting for inclusion in the minimum standard, though another 17% answered that it is not necessary. The digital blood glucose and blood pressure meters reached similar results with 24% rating as required and 20%–22% rating as not necessary. Germicidal lamp is the last device with more than 20% required rating, but with twice as many physicians saying it is not necessary.

The following devices were considered the least useful with more than 1000 “not necessary” ratings: shock wave therapy device, lymphatic massage device, physiotherapy ultrasound device, iFOBT PoCT, NT-pro-BNP device, blood gas analyzer, WIWE mobile ECG device and autoclave. The latter is divisive as 14% of GPs rated it as required. The other seven devices are considered necessary by less than 5%.

**Figure 2.**
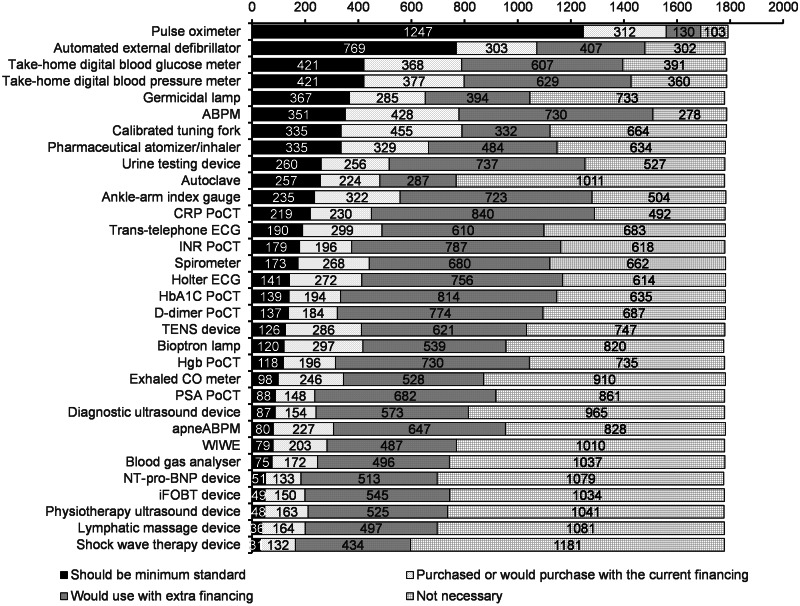
The responding GPs’ perceptions of the usefulness of the devices

The physicians could also select the option that they would use it only with extra financing (either fix or performance-based). These options were especially popular (40%–50%) for the various PoCT and diagnostic devices, likely because of the need for costly accessories and reagents. The detailed answers are depicted in Figure 2.

## Discussion

Taking account of technical requirements of continuously evolving clinical evidence, compared to the existing standard medical device equipment is crucial in order to manage a successful transformation of the PHC system. In line with poor health indicators of Hungarian primary care, the medical device infrastructure was found obsolete and limited compared to clinically evidence-based suggestions especially in single GP-practices, which are dominant in Hungary. GP-practices involved in European and Governmental development projects of the last decade reported a wider range and significantly more intensive utilization of evidence-based technologies, such as ABPM, spirometry, single-lead-ECG for fast screening of atrial fibrillation, or POCT-Lab tests, for example, for HbA1C, CRP and so on. Still, more than half of the devices present in the primary care practices are used only occasionally or not at all. One of the main reasons reported for this is that the high operation costs are not covered by the current financing scheme. A pattern emerges from the findings, showing that development programmes provided the funding for the acquisition of certain medical devices and initially funded the operational costs; however, financing of operational costs was cut-off after a certain time period and long-term financing was not covered. Many respondents agreed that they would consider the more frequent usage of their existing devices if they would receive extra funding for these costs. Although development programmes are key for shifting the currently curative care to a more systematic preventive- and definitive-based primary care, the long-term effects of these programmes need to be reconsidered and the results of such programmes need to be integrated into health policy and health care financing.

GPs involved in GPs-cluster development model programmes showed significantly greater willingness for sharing relatively expensive, extra workforce-demanding technologies, which are considered to be cost-efficient only from at least 10,000 population levels, for example, diagnostic ultrasound device or Nt-pro-BNP POCT-Lab solution. This shows a shift from a single-practice-based mindset to a team-based cooperation in GP cluster and points to a possibly increasing technology usage once GPs form GP clusters under the currently supported state-funded systematic multidisciplinary GPs-cluster model.

## Conclusions

According to national and international guidelines, some of the investigated tools are indispensable for providing state-of-the-art, effective and efficient preventive services, early diagnosis of chronic conditions and adequate care to decrease premature death (Farrance, 2014; Hopstaken *et al.*, 2015; NICE, 2019), the existing minimum standards of medical devices of Hungarian GP-practices need an urgent revision and renewal based on clinical evidence. The indications and recommended processes should be defined and integrated into evidence-based national guidelines. In addition, optimal standards should be defined too, which are not financed, not obligatory, but recommended. The standards need to be revised regularly (yearly). Based on regularly renewed evidence-based standards of medical devices of GPs and GP-cluster services, the establishment of a national registry is required in order to facilitate transparent, efficient, systematic and continuous development of primary health care services. Last but not least targeted incentives should follow the investments into technical development, to result in proper utilization of technologies by trained health care professionals. The aspect of human resource needs of the listed technologies is out of the focus of the article and so is establishing a link between the frequency of technology utilization by GPs and health care indicators of NCDs like cardiovascular disease, diabetes, COPD and so on. Further in-depth research is needed to investigate the reasons why Hungarian GPs are not using medical devices when available to them and how to incentivize and increase the usage to improve health indicators in key chronic disease areas.

## Footnotes

1 Standard minimum devices contain those facilities, which are compulsory for every Hungarian GP practice.
